# Prolonged Hypophosphatemia and Intensive Care After Curative Surgery of Tumor Induced Osteomalacia: A Case Report

**DOI:** 10.3389/fendo.2021.686135

**Published:** 2021-06-03

**Authors:** Eeva M. Ryhänen, Camilla Schalin-Jäntti, Niina Matikainen

**Affiliations:** Endocrinology, Abdominal Center, Helsinki University Hospital and University of Helsinki, Helsinki, Finland

**Keywords:** tumor-induced hypophosphatemia, intensive care, oncogenic osteomalacia, perioperative hypophosphatemia, surgical complications, tumor-induced osteomalacia, fibroblast growth factor 23

## Abstract

**Introduction:**

Rare FGF23-producing mesenchymal tumors lead to paraneoplastic tumor-induced osteomalacia (TIO) presenting with phosphate wasting, hypophosphatemia, chronic hypomineralization of the bone, fragility fractures and muscle weakness. Diagnosis of TIO requires exclusion of other etiologies and careful search for a mesenchymal tumor that often is very small and can appear anywhere in the body. Surgical removal of the tumor is the only definitive treatment of TIO. Surgical complications due to chronic hypophosphatemia are not well recognized.

**Case Description:**

The current case describes severe fragility fractures in a 58-year-old woman, who lost her ability to walk and was bedridden for two years. First, the initial diagnostic laboratory work-up did not include serum phosphorus measurements, second, the suspicion of adverse effects of pioglitazone as an underlying cause delayed correct diagnosis for at least two years. After biochemical discovery of hyperphosphaturic hypophosphatemia at a tertiary referral centre, a FGF23-producing tumor of the mandible was discovered on physical examination, and then surgically removed. Postoperatively, severe hypophosphatemia and muscle weakness prolonged the need for ventilation support, intensive care and phosphate supplementation. After two years of rehabilitation, the patient was able to walk short distances. The tumor has not recurred, and serum phosphate concentration has remained within normal limits during 3.5 years of follow-up.

**Conclusions:**

The case report illustrates knowledge gaps in the diagnostic work-up of rare causes of low bone mass and fragility fractures. Compared to other low phosphate conditions, surgical recovery from TIO-induced hypophosphatemia warrants special attention. Increased alkaline phosphatase concentration may indicate impaired postsurgical recovery due to prolonged hypophosphatemia, underlining the need for proactive perioperative correction of hypophosphatemia.

## Introduction

Tumor-induced osteomalacia (TIO) is a rare paraneoplastic syndrome caused by increased Fibroblast growth factor 23 (FGF23) secretion, typically from a small mesenchymal tumor. In TIO, FGF23 excess drives phosphate wasting and leads to hypophosphatemic oncogenic osteomalacia. Patients typically present with severe symptoms of bone pain, skeletal muscle myopathy, multiple fractures or pseudofractures, skeletal deformities, and impaired quality of life (1, 2).

The diagnosis of TIO is challenging but surgical removal of the phosphaturic mesenchymal neoplasm potentially cures the patient (1, 3). Although the significance of transient phosphate disorders during surgery and intensive care are extensively described, the specific problems associated with chronic hypophosphatemia due to a FGF23-producing tumor in postsurgical patients are poorly described in the literature. The current case underlines the need for careful presurgical planning and active phosphate replacement in severe hypophosphatemia caused by TIO to prevent prolonged need for ventilation assistance, and intensive care.

## Case Description

A 58-year-old female had a three year-history of multiple stress fractures and muscle weakness. Her medical history included well-controlled type 2 diabetes (HbA1c 43 mmol/mol) for nearly 20 years. Her BMI was 39 kg/m^2^ and she was on metformin, long-acting insulin and pioglitazone. She was a current smoker with a smoking history of 27 pack years and her dietary calcium intake was considered normal. Skeletal growth had been normal, and she had not suffered from bone fractures or abnormal skeletal pains previously. She did not have a family history of skeletal, metabolic or hormonal disorders.

**Figure 1:**
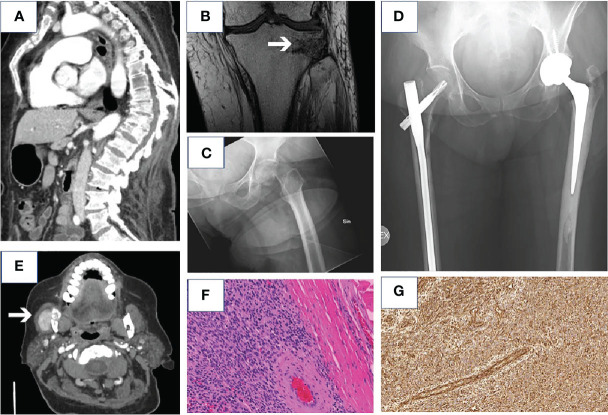
Radiological images of the fragility fractures diagnosed before the diagnosis of TIO. Compression fractures and kyphoscoliosis of the spine in CT **(A)**, MRI scan showing fragility fracture of the lateral condyle of the right tibia **(B)**, X-ray of the fragility fracture of left femoral neck **(C)** and X-ray of pelvis showing arthroplasty of the left hip and osteosynthesis performed after fragility fractures in right femoral shaft and lateral condyle after surgery **(D)**. CT scan of the tumor in the mandible **(E)**, hematoxylin and eosin staining **(F)** (18.4 x) and vimentin staining (positive) (15.9 x) **(G)** of the tumor.

Three years prior to diagnosis the patient presented with multiple fragility fractures and increasing bone pain. Fractures were found in the vertebrae (**Figure 1A**), right femur, sacrum and lateral condyle of the right tibia, and in the left talus. She complained of progressive muscle weakness and diffuse bone pains in her back and lower extremities.

One year later, dual-energy X-ray absorptiometry (DXA) demonstrated osteopenic bone mineral density (T-scores of lumbar vertebrae 3-4 and the femoral neck were -0.8 SD, and -2.3 SD, respectively). Basic biochemistry in primary care was unremarkable (data not shown). Pioglitazone treatment, initiated four years before the first fragility fracture, was suspected to underlie the multiple, low-intensity fractures and therefore discontinued. At this point, type 2 diabetes was considered as another predisposing factor.

During the year before the diagnosis, she suffered from a stress fracture of the left femur with significant dislocation (**Figure 1C**), which was treated with total hip arthroplasty, as well as a stress fracture of the right femur that was treated with an intramedullary nail (**Figure 1D**). Within the next months, she also suffered from stress fractures of the right distal femur, tibia (**Figure 1B**) and humerus.

A year before correct diagnosis of TIO, teriparatide, calcium carbonate (1000 mg daily) and cholecalciferol (20 µg daily) supplementations were initiated to enhance recovery of bone strength after pioglitazone treatment.

The patient was referred to the University Hospital Endocrine Unit. At this time point, the patient had not been able to walk for two years, was bedridden and had lost 30 kg of weight (from 106 to 78kg), and she needed constant pain medication (oxycodone 50 mg, gabapentin 1800 mg and paracetamol 3 grams daily). Biochemical tests revealed hypophosphatemia, combined with high serum FGF23-concentration (C-terminal, EIA-method, MVZ Labor Dr. Limbach & Kollegen, Heidelberg, Germany), inappropriately normal 24-hour urinary phosphate secretion, and increased serum alkaline phosphatase concentration (**Table 1**). Alphacalcidol (0.25 µg twice daily) and phosphate supplementation (Phosphate Sandoz 500 mg twice daily, reduced dose due to diarrhea) were started. Calcium carbonate 1000 mg and cholecalciferol 20 µg daily were continued and teriparatide was discontinued. A bone biopsy from the iliac crest demonstrated osteomalacia. Genetic testing for hypophosphatemia [mutations in the *DMP-1*, *ENPP1*, *FGF23*, *PHEX* and *SLC34A3* genes (4)] was negative. Computed tomography (CT) of the thorax and abdomen revealed no tumors. An additional, complete physical examination revealed a palpable mass in the right mandible. Ultrasound and CT confirmed a tumor with invasive growth in the right mandible (**Figure 1E**). Cytologic analysis of a fine needle aspiration taken from the tumor demonstrated myoepithelial-like cells.

**Table 1:** Biochemical findings at diagnosis and 20 months after surgery.

Parameter (reference range)	At diagnosis	1 month after surgery	16 - 20 months after surgery
Ionized calcium (1.15-1.30 mmol/l)	1.28	1.26	1.22
Phosphate (0.76-1.41 mmol/l)	0.39	1.39	1.09
PTH (15-65 ng/l)	40	73	51
Alkaline phosphatase (35-105 U/l)	489	482	123
Creatinine (50-90 μmol/l)	60	47	60
25-OH-D (> 50 nmol/l)	112	69	83
1,25-(OH)_2_D (52-267 pmol/l)	25	–	132
24h urinary phosphate (20-50 mmol)	12.9	–	8.4
FGF23 (26-110 kRU/l)	2410	279	364
PINP (13-116 μg/l)	188	(578, 6 mo after surgery)	63

PTH, parathyroid hormone; FGF23, fibroblast growth factor 23; PINP, Procollagen type 1 N-propeptide.

After the diagnosis was confirmed, the tumor of the right mandible was operated. Histopathological examination of formalin-fixed and paraffin-embedded slides demonstrated a 2.6 cm phosphaturic mesenchymal tumor with multinucleated, osteoclast-like cells (**Figure 1F**), with minimum tumor free resection margins of 0.5 mm. The Ki-67 proliferation index in hot spots was 10-15%. Immunohistochemical studies showed positive vimentin staining (**Figure 1G**) but negative CD34, EMA, and CKPAN stainings, compatible with a phosphaturic mesenchymal tumor and the diagnosis of TIO (5). The histologic samples were re-evaluated by an expert pathologist in the National Institutes of Health, USA.

Postoperatively, the patient needed prolonged treatment of 10 days in the intensive care unit. Both intravenous and p.o. phosphate supplementations were warranted to correct for severe hypophosphatemia (**Table 2**). After surgery, the concentration of serum ionized calcium remained in the normal range, 1.16-1.25 mmol/l (reference range 1.15-1.30 mmol/l). Administration of calcium carbonate 1000 mg and cholecalciferol 20 µg daily was continued. Due to muscle weakness, the patient was dependent on mechanical ventilation for four days. Thereafter, she needed continuous positive airway pressure (CPAP) through a tracheostomy. During that time, to maintain sufficient blood pressure levels, norepinephrine infusion had to be maintained for one week after surgery. She was on enteral nutrition during the postoperative days 1-11 (1500 kcal daily from third postoperative day). On the third postoperative day, the patient presented with respiratory alkalosis for six hours after fiberoptic bronchoscopy, with a pH of 7.52-7.60 and low/normal pCO2 (3.6-4.9 kPa), and elevated pO2 (13.2-59.8 kPa), base excess (BE) (2.5-5.7 mmol/l) and bicarbonate (HCO^3^) concentrations (27-30 mmol/l) (reference ranges 7.35-7.45, 4.5-6.0 kPa, 9.3-12.3 kPa, -2.5-2.5 mmol/l and 22-24 mmol/l, respectively). From the fourth postoperative day onwards, arterial pH remained within normal range. The patient was decanylated one week postoperatively (**Table 2**). She received subcutaneous short-acting insulin (4-12 units daily) for hyperglycaemia.

**Table 2:** Serum phosphate, ionized calcium and PTH concentrations, phosphate substitution and during 10-day postoperative intensive care.

Number of days after surgery	S-Pi, mmol/l*	Ionized calcium, mmol/l*	PTH, ng/l*	Phosphate substitution (iv.) per 24 hours	Phosphate substitution (po.)	Mode of ventilation support
1 month preoperatively	0.45	1.19	47	none	Phosphate 1000 mg	no
Surgery	not measured	1.27		52 mmol	none	ventilator
Day 1	not measured	1.26		52 mmol	none	ventilator
Day 2	0.27 (morning) 0.91 (evening)	1.24		78 mmol	none	ventilator
Day 3	0.38	1.15		85 mmol	none	ventilator
Day 4	0.59	1.20		92 mmol	Phosphate 500 mg	ventilator^#^
Day 5	0.56	1.21		62 mmol	Phosphate 500 mg	extubated, noninvasive ventilation (CPAP)
Day 6	0.66	1.22		62 mmol	Phosphate 500 mg	CPAP
Day 7	not measured	1.25	69	42 mmol	Phosphate 500 mg	CPAP
Day 8	0.85	1.26		none	Phosphate 1000 mg	CPAP
Day 9	not measured	1.24		none	Phosphate 1000 mg	Oxygen mask
Day 10	1.00	1.28		none	Phosphate 1000 mg	Oxygen mask
Day 30	1.30	1.26	73	none	Phosphate 1000 mg	None

*reference ranges 0.76-1.71 mmol/l, 1.15-1.30 mmol/l, 15-65 ng/l, respectively. ^#^oxandrolone was started and given for one week.

CPAP, Continuous positive air pressure.

Calcium carbonate supplementation of 1000 mg daily remained stable during the pre- and postoperative period.

Phosphate supplementations were administered for eight weeks. The patient received oxandrolone for one week because of extreme weakness and lack of spontaneous respiratory activity in order to improve muscle function and the recovery of lean body mass (6). Skeletal and limb pains relieved soon, and muscle weakness alleviated within the succeeding months. The patient was able to take a few steps after four months of rehabilitation.

**Figure 2:**
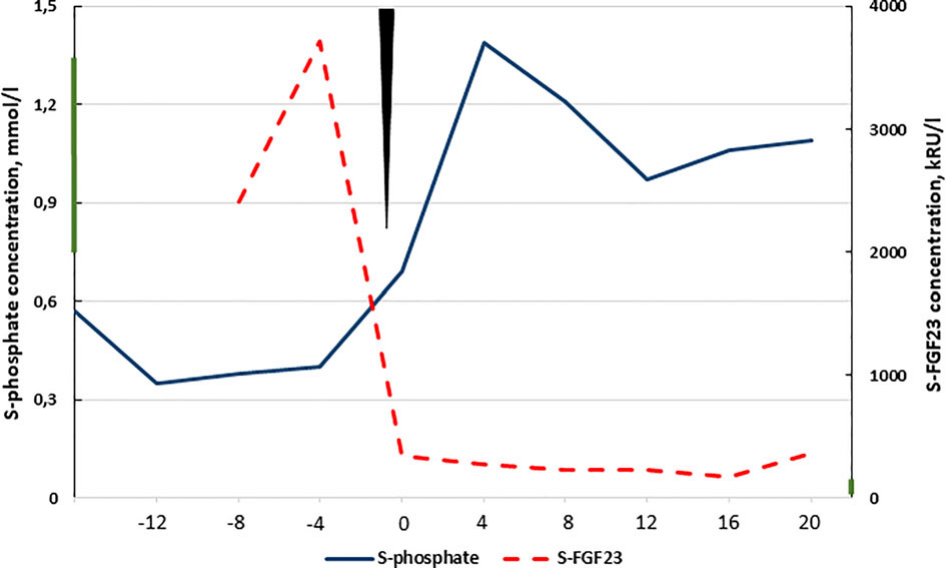
Timeline showing serum phosphate and FGF23 (C-terminus) concentrations at diagnosis and postoperatively. Time of surgery is marked with the black triangle and X-axis presents the time in months relative to surgery. Reference ranges are 0.76-1.41 mmol/l, and 26-110 kRU/l are marked to the y-axes with a bar, respectively.

After two years of rehabilitation, the patient was able to walk 100 meters with a walker and shorter distances without any help. She has not suffered from any further fractures or bone pains. After surgery, serum phosphate concentration has remained normal. Serum FGF23 concentrations decreased immediately after the operation and have remained slightly increased (**Figure 2**). Due to the vertebral fractures, DXA of the spine was unreliable.

## Literature Search

We identified MeSH-terms (https://www.nlm.nih.gov/mesh/meshhome.html) for possible studies reporting postoperative complications during surgery for TIO. The identified MeSH-terms were searched in PubMed/MEDLINE database from 1966 to February 28, 2021. No publications describing postoperative prolonged hypophosphatemia, surgical complications, or prolonged need for intensive care or mechanical ventilation support in TIO patients were found. The electronic search was supplemented by manually searching reference lists of recent review articles that revealed two case reports of postoperative recovery in patients with TIO (1, 3).

## Discussion

Here, we describe a 58-year old patient with TIO, who presented with prolonged postoperative need for phosphate supplements and ventilation support in the intensive care unit because of severe, persistent hypophosphatemia and respiratory muscle weakness. The decrease in postoperative phosphate levels mimicked a transient hungry bone syndrome-like event that resolved after normalization of bone markers including alkaline phosphatase.

According to the literature search, persistent hypophosphatemia complicating the recovery from surgical treatment of TIO has not been described previously. In our patient, the post-surgical prolonged hypophosphatemia and muscle weakness required i.v. and p.o. phosphate supplementation up to eight weeks, despite complete removal of the mesenchymal tumor and later permanent normophosphatemia. According to previous publications, after excision of the mesenchymal tumor, serum phosphate and FGF23 concentrations usually return to normal within the first five post-surgical days, with gradual alleviation of symptoms. During the recovery phase, the skeleton is actively remineralizing and patients may transiently require supplemental calcium to prevent hypocalcemia and secondary hyperparathyroidism (1, 7). Chong et al. (7) describe that the severity of the metabolic bone disease correlates with the serum alkaline phosphatase concentrations, and that kidney phosphate reabsorption may recover slowly. In our patient, severe hypophosphatemia was associated with progressive increase in serum alkaline phosphatase concentrations and bone markers, and loss of muscle strength. The bone biopsy revealed severe osteomalacia, which is a typical histological finding in hyperphosphaturic hypophosphatemia (8). In the present case, a multifactorial background probably best explains hypophosphatemia, including gradual recovery of renal proximal tubular dysfunction and increased phosphate uptake by the skeleton leading to a transient hungry bone syndrome-like decrease in postoperative phosphate levels but the precise mechanisms remains unclear (9). A limitation in our case includes lack of measurements of tubular maximum reabsorption of phosphate and 1,25-(OH)_2_D immediately post-operatively. However, other causes for hypophosphatemia such as alkalosis or the use of norepinephrine or insulin only briefly contributed to hypophosphatemia during the ICU stay and adequate nutrition was maintained to prevent the re-feeding syndrome (10).

Severe hypophosphatemia is a well-known risk factor for delayed postoperative recovery. In our patient, other factors that could have contributed to late weaning from assisted ventilation include smoking, obesity and diabetes. Phosphate is essential for the synthesis of ATP (adenosine triphosphate) and 2,3-diphosphoglycerate, both of which are critical for the regulation of cell metabolism and muscle contractility (11). In intensive care patients, hypophosphatemia is associated with increased morbidity, including longer dependence on mechanical ventilation and prolonged hospitalization. Hypophosphatemia decreases contractility of the diaphragm, left ventricular function, insulin sensitivity, and functions of the central nervous system, which predispose to postoperative complications such as respiratory insufficiency, increased incidence of ventricular tachycardia, impaired glucose metabolism, seizures, and coma (12–17). Respiratory alkalosis, hyperglycemia, insulin and cathecholamines may further worsen hypophosphatemia during intensive care (16–18). These risks are relevant for patients with TIO, in whom the treatment aims at curative surgery.

Although the phosphate concentration normalized within two months, the C-terminal FGF23 concentration remained slightly elevated despite biochemical and physical recovery (**Figure 2**). Previous literature suggests that this may reflect an alteration in FGF23 processing that increases the biologically inactive FGF23 fraction (19).

We performed a literature search to summarize the current knowledge of surgical complications and identified only three case reports of postoperative problems associated with TIO (20–22), which emphasize the need for careful assessment of cardiovascular and respiratory status both pre- and postoperatively. However, in these cases, postoperative recovery was only mildly disturbed. Our patient presented with long-term myopathy and, postoperatively, needed 10 days of treatment in the intensive care unit due to difficulties in weaning from mechanical ventilation and hypotension, despite continuous intravenous and oral phosphate supplementations. After normalization of plasma phosphate, muscle weakness persisted for months and rehabilitation is still ongoing. In patients with TIO, careful planning of the postsurgical management including measurements of alkaline phosphatase and phosphorus concentrations is thus essential.

Fragility fractures are a diagnostic challenge. They are more common after menopause and in patients with type 2 diabetes, both of which increase bone fragility by enhancing the cortical porosity and decreasing the cortical area and bone material strength (23). Glitazone treatment is associated with an augmented risk of fragility fractures, including both upper and lower limb, as well as hip fractures. Both rosiglitazone and pioglitazone appear equally likely to adversely affect bone health (24–26). In our patient, treatment with pioglitazone for 4.5 years before referral to the Endocrine University Hospital Unit was suspected to underlie the multiple fragility fractures. To our knowledge, glitazones are not known to cause osteomalacia in bone histology. In animal studies, histomorphometric analysis of the trabecular bone of the proximal tibia demonstrate increased fat content and number of adipocytes during rosiglitazone administration. In addition, osteoblast activity is decreased and osteoclast activity increased (27, 28). It is unlikely that type 2 diabetes or pioglitazone use alone could have caused the clinical scenario in the present case, which initially was dominated by multiple severe fractures caused by minimal trauma. Speculatively, these factors may have caused additional bone frailty on top of long term TIO.

The current case demonstrates the importance of thorough assessment of basic low-cost laboratory tests in patients with low energy fragility fractures, since the delay in the diagnosis was partly due to lack of determinations of serum phosphate concentrations. A recent large series of 144 patients with TIO demonstrates that 95% of patients are initially misdiagnosed, or characteristic laboratory findings are neglected (29). Importantly, current European guidance for the diagnosis and management of osteoporosis in postmenopausal women (30) recommend measurement of serum phosphate and alkaline phosphatase to exclude a disease which can mimic osteoporosis, such as any type of osteomalacia. In our patient, other differential diagnostic testing included exclusion of vitamin D deficiency, chronic renal and/or hepatic diseases and, genetic hyperphosphaturic diseases due to mutations in the *DMP-1, ENPP1, FGF23, PHEX* and *SLC34A3* genes. In addition to the clinical picture, the age of our patient, the results of biochemical testing, the finding of impaired bone mineralization on bone biopsy, as well as the significantly increased serum FGF23 concentrations were consistent with the diagnosis of TIO (1, 30).

Given their small size, FGF23-producing mesenchymal tumors are difficult to detect with anatomic imaging (radiography, ultrasound, CT and MRI). In our patient, physical examination revealed a tumor in left mandible region that was confirmed by ultrasound. A biopsy was obtained but is not generally recommended because it may lead to tumor seeding (31). Currently, a stepwise approach is recommended, including head to toe functional imaging with Ga-68 DOTATATE PET/CT, the sensitivity of which is better than that of 18F-FDG PET/CT (32). Some centers use selective venous sampling of FGF23, which may be indicated in special situations (33, 34). Most tumors reside in the extremities, while tumors of the maxilla or mandible have been reported less frequently. In a report of 39 patients from Beijing, 56% of the tumors were found in the lower extremities, 5% in the upper extremities, 3% in the hip, 31% in the head, and 5% in the thoracic region (35). Eight of 12 tumors found in the head were located in the mandible or the maxilla. Two tumors were detected on physical examination, and one of them was located in the mandible (35). In another series of 17 Chinese patients, 53% of the tumors were revealed on physical examination, one of which was found in the mandible (36). In the current case, the mesenchymal phosphaturic tumor involving the mandible was detected by palpation, which underlines the importance of thorough basic physical examination.

Phosphaturic mesenchymal tumors are characterized by distinct molecular genetic features with high prevalence of fibronectin 1 (FN1)-FGFR1 fusion gene and less frequently a FN1-FGF1 fusion, which are detected in in 42% and 6% of cases, respectively and may aid the pathologic diagnosis (37). We could not obtain a molecular genetic testing of these fusion gene rearrangements in our case. However, the histologic samples were re-evaluated by an expert pathologist in the National Institutes of Health, USA, to confirm the histological diagnosis.

The current case illustrates knowledge gaps in the diagnostic work-up of rare causes of low bone mass and fragility fractures. Phosphorus was measured only after referral to a tertiary center. Pioglitazone use possibly further worsened the clinical course and certainly delayed the diagnosis of TIO in our patient. We emphasize the need to recognize that severe myopathy, longstanding presurgical hypophosphatemia and elevated alkaline phosphatase concentrations may indicate major difficulties in postsurgical weaning from the ventilator, and delayed recovery. In such cases, perioperative hypophosphatemia should be corrected proactively. In the future, novel FGF23-antagonists may prove useful in the preoperative treatment of severe hypophosphatemia when other approaches fail (38, 39).

## Data Availability Statement

The raw data supporting the conclusions of this article will be made available by the authors, without undue reservation.

## Ethics Statement 

Ethical review and approval was not required for the study on human participants in accordance with the local legislation and institutional requirements. The patients/participants provided their written informed consent to participate in this study.

## Author Contributions

NM and CS-J designed the case report. ER and NM collected the data and NM reviewed the literature. ER, CS-J, and NM drafted and revised the manuscript. All authors contributed to the article and approved the submitted version.

## Funding

The authors declare that this study received funding from independent research grants from Finska Läkaresällskapet (to CS-J) and the Helsinki University Hospital Research Funds (TYH2017138 and TYH2018223 to CS-J) and (TYH TYH2020402 and M1021YLI31 to NM). The funders were not involved in the study design, collection, analysis, interpretation of data, the writing of this article or the decision to submit it for publication.

## Conflict of Interest

The authors declare that the research was conducted in the absence of any commercial or financial relationships that could be construed as a potential conflict of interest.

## Abbreviations

ATP, adenosine triphosphate; BMI, body mass index; CKPAN, Pan-cytokeratin; CPAP, continuous positive airway pressure; CT, computed tomography; DXA, dual-energy absorptiometry; EIA, enzyme immunoassay; FGF23, fibroblast growth factor 23; MRI, magnetic resonance imaging; TIO, tumor-induced osteomalacia.
